# Cross-Sectional Associations of Metabolically Healthy Obesity, Lifestyle Factors, and Steatotic Liver Disease in Adults from the Fels Longitudinal Study

**DOI:** 10.3390/metabo16050299

**Published:** 2026-04-28

**Authors:** Ariana L. Garza, Audrey C. Choh, John Blangero, Cici X. Bauer, Stefan A. Czerwinski, Miryoung Lee

**Affiliations:** 1 School of Public Health, UTHealth Houston, Brownsville, TX 78520, USA; 2 Department of Epidemiology, School of Public Health, UTHealth Houston, Brownsville, TX 78520, USA; audrey.c.choh@uth.tmc.edu (A.C.C.); miryoung.lee@uth.tmc.edu (M.L.); 3 South Texas Diabetes and Obesity Institute, School of Medicine, University of Texas Rio Grande Valley, Brownsville, TX 78520, USA; john.blangero@utrgv.edu; 4 Department of Biostatistics, School of Public Health, UTHealth Houston, Houston, TX 77030, USA; cici.x.bauer@uth.tmc.edu; 5 School of Health and Rehabilitation Sciences, College of Medicine, Ohio State University, Columbus, OH 43210, USA; stefan.czerwinski@osumc.edu

**Keywords:** non-alcoholic fatty liver disease, metabolic dysfunction-associated steatotic liver disease, NAFLD, MASLD, metabolic health, obesity, steatosis

## Abstract

Objective: To examine the associations of metabolic health and obesity phenotypes with liver fat accumulation and hepatic steatosis in adults. Methods: We analyzed 676 non-Hispanic white adults (18–95 years; 55.8% female) from the Fels Longitudinal Study using a cross-sectional design. Participants were classified into metabolically healthy normal weight (MHNW), metabolically healthy obesity (MHO), metabolically unhealthy normal weight (MUNW), and metabolically unhealthy obesity (MUO) phenotypes. Metabolically unhealthy status was defined as the presence of ≥1 metabolic dysfunction, consistent with prior epidemiological definitions; secondary analyses using ≥2 were also performed. Obesity was defined using DXA-derived body fat percentage. Liver fat (%) was quantified using magnetic resonance imaging, and hepatic steatosis was defined as liver fat > 5.56%. Multivariable linear and probit regression models were used to evaluate associations, adjusting for demographic and lifestyle covariates; secondary models additionally examined dietary intake. Results: Mean liver fat was 5.95% (SE = 0.23), and steatosis was present in 29.8% of participants. Compared to MHNW individuals, liver fat was significantly higher in MHO (mean 3.77% vs. 2.67%), MUNW (4.63%), and MUO (8.47%) phenotypes. After covariate adjustment, liver fat was 33.8% (95% CI: 13.7–57.5%) higher in MHO, 28.1% (10.1–49.0%) higher in MUNW, and 113.0% (85.3–144.7%) higher in MUO relative to MHNW. Corresponding increases in steatosis probabilities were observed across phenotypes. No individual dietary component or dietary pattern was significantly associated with liver fat after adjustment. Conclusions: Metabolically healthy obesity was associated with higher liver fat and steatosis probability compared with metabolically healthy normal weight, with levels comparable to metabolically unhealthy normal weight individuals. These findings suggest that the absence of overt metabolic abnormalities does not necessarily indicate a metabolically benign state with respect to liver fat accumulation. Given the cross-sectional design, these results should be interpreted as associations rather than causal relationships.

## 1. Introduction

Obesity is a major risk factor for non-alcoholic fatty liver disease (NAFLD), newly referred to as metabolic dysfunction-associated steatotic liver disease (MASLD), as well as other adverse health outcomes, including cardiovascular disease (CVD), type 2 diabetes (T2D), and certain kinds of cancer [1]. Poor metabolic health, characterized by factors such as insulin resistance and dyslipidemia, has also been shown to predict NAFLD [2,3]. Increasing attention has been given to the combined effects of obesity and metabolic health, particularly to the concept of metabolically healthy obesity (MHO). Some studies suggest that individuals with MHO may be at a lower risk for such health outcomes compared to those with metabolically unhealthy obesity (MUO), regardless of the criteria used to define metabolic health itself [4,5,6,7]. However, this remains controversial, as evidence indicates that MHO may represent a transient state rather than a stable, benign phenotype [4,6].

Emerging evidence suggests that individuals with MHO may still exhibit elevated cardiometabolic risk compared with metabolically healthy individuals with normal weight [8]. Even in the absence of overt metabolic abnormalities, subtle physiological changes such as ectopic fat deposition may already be present. In particular, hepatic fat accumulation has been proposed as an early manifestation of metabolic dysfunction that may precede clinically overt metabolic syndrome [8]. These findings highlight the importance of evaluating early indicators of liver disease and overall metabolic risk within obesity phenotypes traditionally considered as low risk.

Several studies have already examined the relationship between metabolic health, obesity and NAFLD. In a U.S. cohort of predominantly white individuals with MHO undergoing bariatric surgery, 35% had undiagnosed NAFLD and 10% had advanced fibrosis [9]. In a prospective study of metabolically healthy Korean adults, higher body mass index (BMI) was associated with increased incidence of NAFLD over 4.5 years, regardless of changes in metabolic status [10]. Similarly, BMI was associated with worsening NAFLD fibrosis scores over time, and approximately 70% of individuals classified as MHO at baseline eventually transitioned to a metabolically unhealthy state [11].

Lifestyle factors, including diet and physical activity, are also known to influence both obesity and metabolic health and may contribute to NAFLD risk [12,13,14]. However, population-based evidence remains limited and findings are inconsistent. For example, protein intake has been associated with liver fat in small studies [14], whereas physical activity has not shown consistent associations after adjusting for adiposity. In larger cohorts such as the UK Biobank, higher intake of dietary fat, particularly saturated and animal fats, has been linked to increased liver fat, although these associations may vary by sex [15].

Despite these numerous findings, the relationship between metabolic health phenotypes and the very early stages of NAFLD has not yet been extensively evaluated using precise, non-invasive measures of liver fat. Magnetic resonance imaging (MRI), particularly proton density fat fraction (PDFF), provides accurate quantification of liver fat and correlates strongly with histological assessments [16,17,18]. This approach allows for the detection of early hepatic fat accumulation, defined as steatosis (fat infiltration in more than 5.56% of hepatocytes) [19].

In addition to imaging-based assessments, metabolic health is also influenced by biochemical and physiological factors not fully captured by standard clinical definitions. Biomarkers such as triglycerides, high-density lipoprotein cholesterol, glucose, and liver enzymes contribute to metabolic dysfunction and may provide further insight into early alterations associated with liver fat accumulation [20]. Furthermore, differences in body composition, including fat distribution and lean body mass, may contribute to the observed heterogeneity in metabolic health phenotypes beyond what is captured by BMI alone [20].

Variability in findings across studies may also reflect differences in how obesity and metabolic health are assessed and defined. Most studies rely on BMI, which does not fully capture body composition. In contrast, body fat measured by dual-energy X-ray absorptiometry (DXA) is considered a more accurate indicator of true body adiposity [21,22].

Based on this evidence, we hypothesized that individuals with metabolically healthy obesity would exhibit greater liver fat accumulation compared with metabolically healthy normal-weight individuals, and that these associations would persist after accounting for lifestyle factors.

## 2. Materials and Methods

### 2.1. Study Design and Participants

This study was a cross-sectional analysis of existing data from the Fels Longitudinal Study (FLS), a family-based study of growth, development, and body composition that began in 1929 in Yellow Spring, Ohio. A total of 704 adult participants with at least one MRI-assessed liver fat content measurement acquired between August 2012 and June 2015 were selected for inclusion. Participants were asked to sign study consent forms at each visit under institutional review board (IRB) approval. All recruitment took place at Wright State University. FLS participants were deemed eligible for inclusion in this portion of the study if they did not have any contraindications for an MRI assessment or a history of chronic liver disease. This data analysis was conducted under IRB approval at UTHealth: HSC-SPH-17-0262. We excluded participants with a history of heavy alcohol consumption, defined as having >4 drinks on any day for men and >3 drinks on any day for women (*n* = 8).

### 2.2. Measurements

The primary outcome, liver fat, was assessed using a Magnetom-Avanto 1.5 Tesla whole body scanner with Syngo MR-B15 software (Siemens Healthineers, Erlangen, Germany) [23]. All participants were re-screened for MRI contraindications prior to undergoing liver fat content assessment. Breath-holding maneuvers were utilized to limit motion artifacts, while 6 mm axial slices of the liver were obtained. Scan data were analyzed using specially designed image analysis software and conducted by a single, highly trained analyst with over 11 years of experience in MR analysis. A modification of the Dixon method was used to quantify liver PDFF [24]. The validity of liver fat content assessed through the modified Dixon MRI method was compared to proton magnetic resonance spectroscopy (H-MRS) assessments and found to be highly correlated (*r* = 0.9936) [24].

Liver fat content was also dichotomized for the presence of steatosis (yes or no) based on the cut-point value of greater than 5.56%, corresponding to the 95th percentile of the distribution of hepatic triglyceride content (HTGC) in healthy participants from the Dallas Heart Study. These threshold values have been demonstrated to be highly correlated with the gold standard biopsy analysis for detecting steatosis [19].

Body composition was assessed by DXA, using a Hologic Discovery-A densitometer, following the manufacturer’s protocol [25]. The total body scan was analyzed to yield measures of bone, fat, and lean tissue for total body and specific body regions. The precision and reliability of DXA have been well documented [21,22]. Anthropometric data collected also included weight (kg), stature (cm), and circumferences of the abdomen and hip (cm) following standardized protocols [26]. Seated, resting brachial systolic (SBP) and diastolic blood pressure (DBP) readings were obtained through a standardized protocol using a mercury sphygmomanometer.

Metabolic health blood assay data used in this study included assessments of fasting glucose, insulin, triglycerides, and high-density lipoprotein-cholesterol (mg/dL), all measured using standard laboratory practices at a commercial laboratory (LabCorp). Demographic variables of age, sex (male/female), habitual physical activity score (1–5, with 5 as the highest level of habitual physical activity) using Baecke’s physical activity questionnaire [27], and information on smoking and drinking habits collected at the time of the MRI assessment were included as covariates. Self-administered and semi-quantitative Food Frequency Questionnaires (FFQ) [28] were collected and analyzed at the Nutrition Questionnaire Service Center at Harvard University for nutrient intake and food consumption. Dietary intake data were available for 613 participants, out of which 387 (63.1%) were collected at study visits prior to the MRI measurement of liver fat.

### 2.3. Obesity and Metabolic Health Assessment

Obesity was defined as %BF measured by DXA greater than 25% in men or 32% in women, based on the 2012 guidelines of the American Society of Bariatric Physicians [29]. As a secondary analysis, the models were repeated using an alternative definition of obesity based on the more clinically available BMI cutoff of ≥30 kg/m^2^, as per the World Health Organization’s standards [30]. We adapted the definition of metabolic health proposed by Wildman et al. [31], which applied similar cardiometabolic risk factor cutoffs in NHANES. Metabolically unhealthy status was defined as a participant having at least one of the following five metabolic dysfunctions: (1) systolic blood pressure (SBP) ≥ 130 mmHg, diastolic blood pressure (DBP) ≥ 80 mmHg, treatment for hypertension (HTN), or physician diagnosed HTN; (2) fasting glucose (FG) ≥ 100 mg/dL, treatment for high glucose, or physician diagnosed T2D; (3) high-density lipoprotein (HDL-C) < 40 mg/dL in men or <50 mg/dL in women; (4) triglyceride (TG) level ≥ 150 mg/dL or lipid lowering treatment; or (5) HOMA-IR > sex-specific 90th percentile [32,33]. This definition of metabolically unhealthy status as the presence of ≥1 metabolic abnormality was selected to capture early metabolic dysfunction and align with prior epidemiological studies examining metabolic health phenotypes. To assess the robustness of this definition, secondary analyses using a more stringent definition requiring ≥2 metabolic abnormalities were also performed.

**Figure 1:**
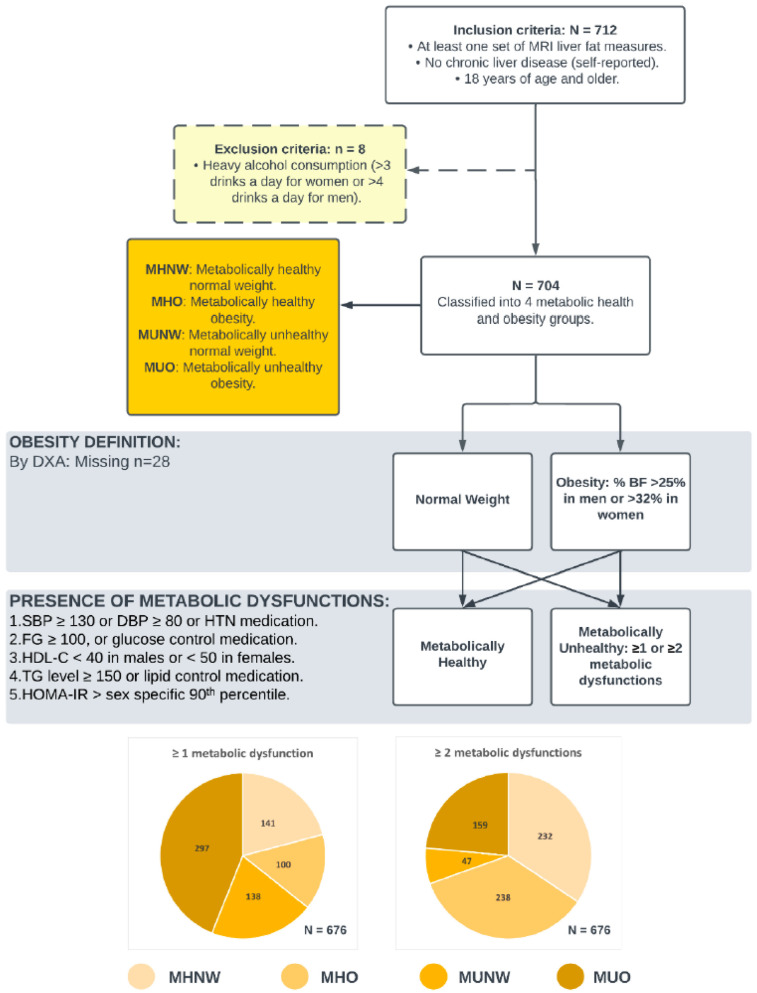
Sample selection and data grouping flow diagram. Abbreviations: MRI, magnetic resonance imaging; MHNW, metabolically healthy normal weight; MHO, metabolically healthy obesity; MUNW, metabolically unhealthy normal weight; MUO, metabolically healthy obesity; DXA, dual energy x-ray absorptiometry; BMI, body mass index; SBP, systolic blood pressure; DBP, diastolic blood pressure; HTN, hypertension; T2D, type 2 diabetes; FG, fasting glucose; HDL-C, high-density lipoprotein cholesterol; TG, triglycerides.

Study participants were then grouped into four metabolic health and obesity phenotypes: metabolically healthy normal weight (MHNW), metabolically healthy obesity (MHO), metabolically unhealthy normal weight (MUNW) and metabolically unhealthy obesity (MUO), resulting in 141, 100, 138, and 297 participants in each phenotype, respectively. Using the alternative definition of metabolic health with ≥2 metabolic abnormalities resulted in 232, 238, 47, and 159 participants for the MHNW, MHO, MUNW, and MUO phenotypes, respectively. A total of 28 participants had missing DXA body fat assessments and were, therefore, excluded from the regression analyses. Figure 1 provides a summary of the data handling process and compares phenotype frequencies by each of the two metabolically unhealthy definitions.

### 2.4. Statistical Analysis

Descriptive clinical and dietary characteristics of study participants, overall and by metabolic health and obesity phenotypes, were computed using SAS v. 9.3 [34]. Generalized linear models using PROC GLM and PROC GLIMMIX procedures with post hoc pairwise comparisons were employed to assess whether there was a significant difference in individual trait means or frequencies between the different phenotypes. In PROC GLIMMIX, family structure was modeled as a random effect to account for relatedness.

Liver fat content (%) was log-transformed. To account for consanguinity in the study sample, we employed the mixed-effect modeling procedures included in the Sequential Oligogenic Linkage Analysis Routines (SOLAR; Texas Biomedical Research Institute, San Antonio, TX, USA; version not specified) software [35], automatically adjusting for an identity-by-descent matrix of interrelatedness measures within all study participant pairs. To model liver fat content (on the log scale), we used multivariable linear regression models, and to model the binary steatosis outcome, we used probit models. Both analyses were performed using MHNW as the reference phenotype. Covariates were selected based on their established associations with liver fat accumulation and metabolic health in prior literature. Age and sex were included to account for demographic differences, while smoking status (current, yes/no), physical activity score and alcohol consumption (number of drinks/day) were included as lifestyle-related factors known to influence liver fat and metabolic risk. Additional metabolic variables such as waist circumference and insulin resistance were not included in the primary models due to potential collinearity with the metabolic health phenotype definitions. A quadratic term for age (age^2^) and a corresponding interaction term with sex (age^2^ × sex) were also included to account for non-linear associations. To facilitate model interpretation, the continuous covariates of age and physical activity score were mean-centered prior to modeling. We used the same regression models on the alternative definition for metabolically unhealthy of ≥2 metabolic dysfunctions for phenotype grouping, and used the alternative definition of obesity based on BMI as a secondary analysis. Statistical significance was assessed with alpha (α) < 0.05.

To further explore the potential contribution of diet to liver fat accumulation across metabolic health and obesity phenotypes, we also conducted secondary analyses incorporating dietary intake variables. First, individual macronutrient intake (total energy, carbohydrates, protein, total fat, saturated fat, monounsaturated fat, polyunsaturated fat, animal fat, and vegetable fat) was modeled separately as fixed covariates in the SOLAR mixed-effects models. Second, to reduce multicollinearity and identify underlying dietary patterns, principal component analysis (PCA) was performed on the standardized macronutrient intake variables. The first three principal components, cumulatively explaining over 95% of dietary intake variance, were included in separate models as covariates, along with interaction terms by phenotype. All dietary models adjusted for the same covariates as the main models and were interpreted as exploratory. Dietary data were not available for all participants due to incomplete food frequency questionnaire responses. Analyses including dietary variables were therefore conducted in a subset of participants with available data. The proportion of missing dietary data is reported, and this limitation is considered in the interpretation of the results.

## 3. Results

Table 1 presents the demographic, clinical, lifestyle and nutritional characteristics of participants by the four metabolic health and obesity phenotypes defined by ≥1 metabolic dysfunction and %BF. Participants with metabolically healthy phenotypes (MHNW and MHO) were significantly younger than those of metabolically unhealthy phenotypes (MUNW and MUO), and there was no significant difference in age between the two metabolically unhealthy phenotypes. All phenotypes showed significantly different sex distributions, with a higher percentage of males in both normal weight phenotypes. Compared to the MHNW phenotype, all other phenotypes had significantly higher weight, BMI, and waist circumference measures as well as a greater percentage of obesity based on BMI ≥ 30 kg/m^2^ and abnormal waist measurements (>102 cm for men or >88 cm for women). Not surprisingly, both metabolically healthy phenotypes (MHNW and MHO) had a significantly better cardiometabolic risk profile than did the metabolically unhealthy phenotypes. Both normal weight phenotypes had greater physical activity scores compared to the phenotypes with obesity. There was no significant difference in current tobacco use or alcohol use among the four phenotypes. None of the dietary nutrient components differed significantly across the four phenotypes, although a marginal difference was observed in carbohydrate intake (*p* = 0.0732). Notably, metabolically unhealthy groups were older than metabolically healthy groups. Although age was included as a covariate in all models, these differences may still influence the observed associations and should be considered when interpreting the results.

The overall average liver fat content was 5.95% (SE = 0.23), and liver steatosis was present in 210 participants (29.8%). Participants with the MUO phenotype had significantly greater liver fat content and steatosis (%) than those with other phenotypes. Both the MHO and MUNW phenotypes had significantly greater levels of liver fat content, log-transformed liver fat content, and steatosis (%) compared to the MHNW phenotype. There was no statistically significant difference between the MHO and MUNW phenotypes in log-transformed liver fat content and frequency of steatosis. It is important to note that individuals with metabolically healthy obesity exhibited significantly higher liver fat levels compared with metabolically healthy normal-weight individuals, suggesting that the absence of overt metabolic abnormalities does not necessarily correspond to a metabolically benign state with respect to hepatic fat accumulation.

Table 2 displays the estimated regression coefficients, with MHNW as the reference phenotype, while adjusting for age, sex (and their interactions), physical activity score, smoking, and alcohol consumption. For the linear regression of log-transformed liver fat content, statistical significance was found for all three metabolic health and obesity phenotypes, sex, the interaction of age and sex, age^2^, and physical activity score. After adjusting for covariates, participants with the MHO phenotype had (e^β=0.291^) = 1.33 times the liver fat content, or 33.8% (95% CI = 13.7–57.5%) greater liver fat content than the MHNW phenotype. Participants with the MUNW phenotype had 28.1% (10.1–49.1%) greater liver fat, and those with the MUO phenotype had 113.0% (85.4–144.7%) greater liver fat than those with the MHNW phenotype after adjusting for covariates.

The probit regression for the probability of steatosis also yielded significant regression coefficients for all three metabolic health and obesity phenotypes, sex, age × sex, and age^2^ × sex. Based on these parameter estimations, for non-smoking, non-drinking, average-aged males (50.5 years) with an average physical activity score (2.4), the probability of steatosis for the MHO, MUNW, and MUO groups increased by 0.27 (95% CI, 0.08–0.49), 0.26 (0.09–0.46), and 0.60 (0.41–0.75), respectively (*p* = 0.000), compared to the MHNW phenotype (0.08; 95% CI, 0.03–0.19). Similarly, for non-smoking, non-drinking, average-aged females (51.7 years) with an average physical activity score (2.1), being of the MHO, MUNW, or MUO phenotype was associated with an increase in steatosis probability of 0.17 (0.05–0.36), 0.16 (0.05–0.33), and 0.48 (0.29–0.67), respectively (*p* = 0.000) from that of the MHNW (0.03; 95% CI, 0.01–0.09). These findings suggest potential sex-specific differences in the magnitude of associations between metabolic health phenotypes and steatosis probability, although the overall pattern of increased risk across phenotypes was consistent in both males and females.

Table 3 presents the linear and probit regression coefficients for log-transformed liver fat content and the probability of steatosis by the metabolic health and obesity phenotypes created using ≥2 metabolic dysfunctions to define metabolically unhealthy, but otherwise using the same statistical analyses and covariates for adjustment as in the previous models. Significant regression coefficients also were found in both the linear and probit models for all three metabolic health and obesity phenotypes, albeit with greater effect sizes when compared to the previous models (Table 2) using ≥1 metabolic dysfunction(s) to define metabolically unhealthy. Compared to the MHNW phenotype, liver fat content was 41.9% (95% CI, 26.2–59.6%) greater in the MHO phenotype, 53.3% (26.7–85.3%) greater in the MUNW phenotype, and 156.2% (124.3–192.8%) greater in the MUO phenotype. Similarly, the probability of steatosis in average males (as described above) increased by 0.32 (95% CI, 0.18–0.46), 0.36 (0.18–0.54), and 0.63 (0.51–0.73) in the MHO, MUNW, and MUO phenotypes, respectively (*p* = 0.000), from that of the MHNW (0.15; 95% CI, 0.08–0.25); and in females, it increased by 0.24 (0.12–0.37), 0.27 (0.12–0.46) and 0.57 (0.42–0.70), respectively, from that of the MHNW (0.07; 0.03–0.13).

In exploratory models adjusting for individual nutrition components, no single macronutrient was significantly associated with liver fat after covariate adjustment. Animal fat intake showed a marginal positive association (*p* = 0.087), but other nutrients, including total energy and carbohydrates, were non-significant (Supplementary Table S1).

PCA revealed three dietary patterns: PC1 represented the overall high intake across all nutrients, PC2 was characterized by high animal and saturated fat relative to vegetable and polyunsaturated fat, and PC3 loaded more strongly on protein and carbohydrate variables. Of these, PC2 was marginally associated with liver fat content (*p* = 0.069), but no significant interactions were observed between PCs and phenotype group.

Across all models, inclusion of dietary variables, whether individually, as a composite score or as PCA-derived components, did not appreciably alter the strength or significance of the associations between metabolic phenotypes and liver fat content. The lack of significant associations between individual dietary components and liver fat after adjustment may reflect the complexity of dietary influences on metabolic health, which are not fully captured by individual macronutrient analyses. It is also possible that the cross-sectional design and incomplete dietary data reduced the ability to detect such associations.

**Figure 2:**
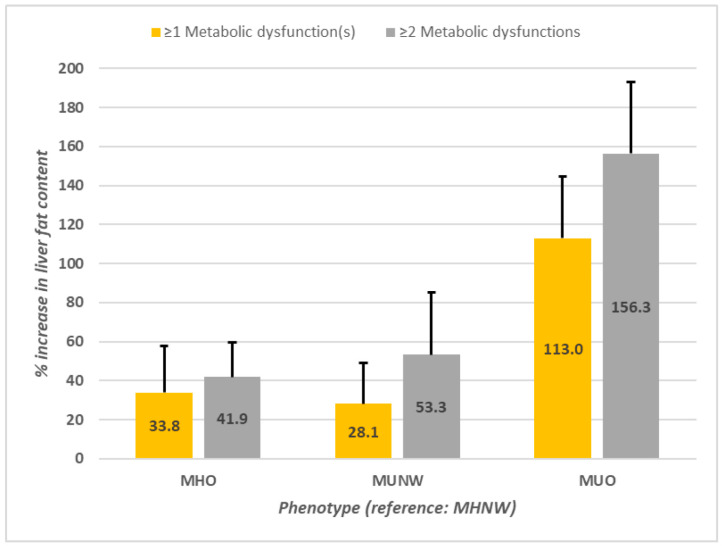
Percent increase in liver fat content associated with each metabolic health and obesity phenotype in reference to the MHNW phenotype, contrasting two different definitions of metabolically unhealthy. Yellow bars represent groups formed using ≥1 metabolic dysfunction to define metabolic health; grey bars represent groups formed using ≥2 metabolic dysfunctions to define metabolic health. Abbreviations: MHNW, metabolically healthy normal weight; MHO, metabolically healthy obesity; MUNW, metabolically unhealthy normal weight; MUO, metabolically unhealthy obesity. Error bars represent 95% confidence intervals.

**Figure 3:**
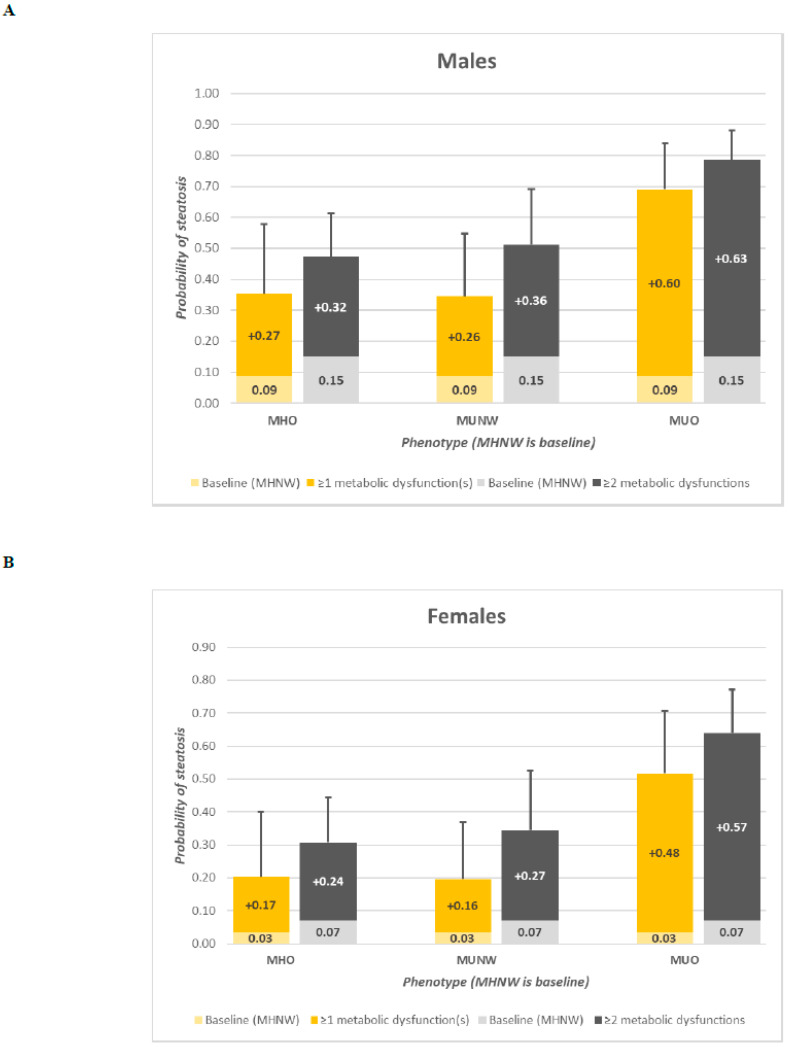
Incremental effects of predicted probability of steatosis associated with each metabolic health and obesity phenotype from that of the MHNW phenotype in (**A**) Average-aged males with average physical activity, no smoking or drinking and (**B**) Average-aged females with average physical activity, no smoking or drinking. Yellow bars represent groups formed using ≥1 metabolic dysfunction to define metabolic health; grey bars represent groups formed using ≥2 metabolic dysfunctions to define metabolic health. Abbreviations: MHNW, metabolically healthy normal weight; MHO, metabolically healthy obesity; MUNW, metabolically unhealthy normal weight; MUO, metabolically unhealthy obesity. Error bars represent 95% confidence intervals.

Figure 1 shows the percentage increases in liver fat content associated with belonging to those with MHO, MUNW, or MUO phenotypes in reference to the MHNW phenotype and the contrasts between the use of the two different definitions of metabolically unhealthy to generate the phenotypes, adjusting for covariates listed in Table 2 and Table 3. Similarly, Figure 2 displays the incremental effects associated with each of the phenotypes in baseline reference of the MHNW among non-smoking, non-drinking males and females of average age and average physical activity scores and the contrast between the use of the two different definitions of metabolically unhealthy to generate the phenotypes. Figure 3 provides a summary of the data handling process and compares phenotype frequencies by each of the two metabolically unhealthy definitions. In a secondary analysis (Supplementary Tables S2 and S3), in which obesity is defined using the more conventional cutoff point of BMI ≥ 30 kg/m^2^, significant regression coefficients for both the linear and probit models were also found for all three metabolic health and obesity phenotypes, regardless of which definition of metabolically unhealthy was used.

## 4. Discussion

In this study, we examined the associations between metabolic health, obesity phenotypes, lifestyle factors, and liver fat accumulation. We found that individuals with metabolically healthy obesity had significantly higher liver fat content compared with metabolically healthy normal-weight individuals, with levels comparable to those with metabolically unhealthy normal weight. Additionally, metabolically unhealthy obesity was associated with the highest levels of liver fat and steatosis probability. These associations remained significant after adjustment for demographic and lifestyle factors and were not substantially influenced by dietary variables.

These findings contribute to the ongoing debate regarding the clinical relevance of metabolically healthy obesity by demonstrating that individuals classified as such already exhibit increased liver fat accumulation compared with metabolically healthy normal-weight individuals. This supports the notion that metabolically healthy obesity may not represent a truly benign phenotype. The prevalence of steatosis (29.8%) in this population is comparable to that reported in recent studies of non-Hispanic whites in the United States using the United States Fatty Liver Index (32%) [36] and the MRI-based Dallas Steatosis Index (33%) [37]. We found that 25.2% of individuals with obesity, defined by DXA-derived %BF, were metabolically healthy (MHO); these individuals had significantly greater liver fat content than those with the MHNW phenotype and were not significantly different from those with the MUNW phenotype in terms of liver fat content. Both metabolically unhealthy phenotypes had significantly greater liver fat deposition than metabolically healthy phenotypes, and the MUO phenotype had the highest levels of liver fat and the highest predicted probability of hepatic steatosis among all four phenotypes.

Epidemiological studies have shown that liver fat may be an important determinant of metabolic health in men and women with obesity, as it has been found to be significantly associated with IR, T2D, and CVD [38,39,40]. For instance, in a sample of 314 white adults, insulin-sensitive individuals with obesity (BMI ≥ 30 kg/m^2^) had significantly lower liver fat than insulin-resistant individuals with obesity and had similar amounts of liver fat as overweight but otherwise metabolically healthy participants, highlighting the role of liver fat in metabolic health among individuals with MHO [41]. In our study, individuals with MHO showed liver fat levels significantly greater than those with MHNW but comparable to those with MUNW, which strengthens the likelihood of liver fat playing an important role in the transition between the metabolically healthy and metabolically unhealthy states. These findings are consistent with prior longitudinal studies indicating that metabolically healthy obesity is often a transient condition and may still be associated with elevated cardiometabolic risk [4,6,42]. The present results extend this literature by demonstrating that differences in liver fat accumulation are already evident at earlier stages, even before the development of overt metabolic dysfunction. This observation also aligns with previous research that positions hepatic fat accumulation as an early physiological alteration in the progression of metabolic dysfunction [20].

The difference in the effect sizes of phenotype membership on liver fat content and probability of steatosis when using the presence of at least two metabolic dysfunctions to define the metabolically unhealthy, rather than at least one metabolic dysfunction, is indicative of a dose–response effect in the association between poor metabolic health and both liver fat accumulation and increasing probabilities of steatosis. A consistent pattern of liver fat accumulation also remained when utilizing the alternative BMI cutoff point to classify obesity (Supplementary Table S3), corroborating that MHO is associated with significantly greater levels of liver fat content and a probability of steatosis compared with the MHNW phenotype, regardless of how obesity is defined.

In addition, metabolic health phenotypes may be influenced by underlying biochemical and physiological differences not fully captured by standard clinical definitions. Biomarkers such as triglycerides, HDL-C, glucose, and liver enzymes have been shown to play important roles in metabolic dysfunction and may provide further insight into early alterations associated with liver fat accumulation [38,39,40]. Differences in body composition such as fat distribution and lean mass may also contribute to the heterogeneity observed in metabolic health phenotypes, helping to explain variability in liver fat accumulation among individuals with similar BMI [29].

In secondary analyses, we explored whether dietary intake contributed to the observed differences in liver fat content across metabolic health and obesity phenotypes. While no single nutrient emerged as a strong independent predictor of liver fat (%) or hepatic steatosis, marginal positive associations were observed for certain variables, such as animal fat intake. Additionally, PCA was used to reduce multicollinearity and identify underlying dietary patterns; one component suggestive of higher saturated and animal fat intake relative to plant-based fats showed a weak association with liver fat content, similar to the results from the UK Biobank study [15]. However, in all models, interaction terms with metabolic health and obesity phenotypes were not significant, suggesting that dietary intake does not substantially modify the relationship between phenotype and liver fat. These exploratory findings align with the literature suggesting diet may contribute to hepatic steatosis risk, but that the phenotype-liver fat associations observed are not strongly confounded or explained by dietary intake alone. Future work using more granular dietary data or longitudinal designs may help clarify the role of diet in metabolic transitions and hepatic fat accumulation. These findings also highlight the limitations of assessing diet using self-reported food frequency questionnaires and suggest that broader dietary patterns or long-term exposures may be more relevant to liver fat accumulation than individual nutrient components alone.

Sex differences are known to influence both obesity-related metabolic dysfunction and liver fat accumulation. In the present study, sex was included as a covariate and interaction term in all the models, and predicted probabilities of steatosis were presented separately for males and females. While the overall pattern of associations between metabolic health phenotypes and liver fat was consistent across sexes, differences in magnitude were observed. These findings suggest that biological and physiological differences between males and females may influence the relationship between metabolic health and hepatic fat accumulation. However, the study was not specifically designed to conduct fully stratified sex-specific analyses, and further research is needed to better characterize these differences.

Our study has several limitations. First, the cross-sectional design precludes causal inference and limits the ability to establish temporal relationships between metabolic health and obesity phenotypes and liver fat accumulation or steatosis risk. As such, the directionality of these associations cannot be determined. Second, the definition of metabolically unhealthy status as the presence of ≥1 metabolic abnormality may represent a more inclusive classification approach; however, sensitivity analyses using a more stringent definition (≥2 abnormalities) yielded consistent results. Third, some dietary data were collected at visits prior to the MRI-based liver fat assessment, which may introduce temporal misalignment between exposure and outcome and potential measurement error. Additionally, dietary data were not available for all participants, and analyses were conducted in a subset of the sample, which may reduce statistical power and increase the potential for bias. Finally, the study sample consists primarily of non-Hispanic white individuals; therefore, the generalizability of our findings to other racial and ethnic populations may be limited.

Despite these limitations, our study addresses an important gap in knowledge by showing that MRI-derived measures of liver fat deposition in adults with MHO are significantly greater than those of MHNW participants and highlights the potential use of precise liver fat content assessments to characterize cardiometabolic risk among individuals with obesity. Our study is also the first to examine the effect of metabolic health and obesity phenotypes on precise measures of liver fat content while adjusting for the effect of familial clustering, adding an important layer to understanding this complex disease process in this population [43].

Current obesity treatment guidelines do not differentiate individuals with MHO from those with MUO and are limited to the application of similar treatment plans, including lifestyle interventions [44]. Further, there is no consensus regarding which cardiometabolic risk definition should be used to distinguish the metabolically healthy from the metabolically unhealthy. Depending on the definitions of obesity and metabolic health used and the population characteristics, considerable variability in the prevalence of MHO exists [4,5,6,7,31,41,45]. The difference between previous studies and ours may lie in the definition of metabolic health, which includes insulin resistance, and a more direct and precise assessment of obesity through DXA scans. Our study, however, also demonstrates that, regardless of which criteria are used for obesity or metabolic health, individuals with obesity but who are otherwise considered metabolically healthy are still at a greater risk for hepatic steatosis than those with normal weight. As such, further studies are warranted to elucidate the complex interplay that exists within metabolic health, adiposity, and liver fat accumulation, taking into consideration longitudinal effects, genetic influences, and gene-by-environment interactions.

## 5. Conclusions

In conclusion, metabolic health and obesity phenotypes are significantly associated with liver fat accumulation. Individuals with metabolically healthy obesity exhibited higher liver fat levels and steatosis probability when compared with metabolically healthy normal-weight individuals. These findings suggest that metabolically healthy obesity may not represent a benign phenotype with respect to liver fat accumulation. Further longitudinal studies are needed to clarify causal relationships as well as the progression of metabolic dysfunction.

## Figures and Tables

**Table 1:** Demographic and clinical characteristics by metabolic health and obesity phenotype.

	**Full Sample** **(N = 704)**	**By Metabolic Health and Obesity Phenotype; (N = 676)**				**Overall *p*-Value**
		**MHNW** **(n = 141)**	**MHO** **(n = 100)**	**MUNW** **(n = 138)**	**MUO** **(n = 297)**	
Age (years)	51.14 (0.69)	39.16 (1.43) ^a^	45.11 (1.69) ^b^	54.68 (1.44) ^c^	57.08 (0.98) ^c^	<0.0001
Sex (male)	311 (44.18)	76 (53.9) ^a^	14 (14) ^b^	107 (77.54) ^c^	94 (31.65) ^d^	<0.0001
Liver fat content (%)	5.95 (0.23)	2.67 (0.46) ^a^	3.77 (0.55) ^a,b^	4.63 (0.47) ^b^	8.47 (0.32) ^c^	<0.0001
Log-transformed liver fat	1.44 (0.03)	0.90 (0.05) ^a^	1.15 (0.06) ^b^	1.30 (0.05) ^b^	1.81 (0.04) ^c^	<0.0001
Steatosis (yes)	210 (29.83)	4 (2.84) ^a^	14 (14.0) ^b^	30 (21.74) ^b^	145 (48.82) ^c^	<0.0001
Adiposity Measures						
Weight (kg)	80.68 (0.71)	68.10 (1.32) ^a^	78.27 (1.57) ^b^	76.70 (1.33) ^b^	85.69 (0.91) ^c^	<0.0001
BMI (kg/m^2^)	27.59 (0.21)	22.51 (0.35) ^a^	27.69 (0.42) ^b^	24.84 (0.35) ^c^	30.43 (0.24) ^d^	<0.0001
% Body Fat (676)	29.92 (0.34)	20.92 (0.45) ^a^	34.91 (0.54) ^b^	22.12 (0.46) ^a^	36.15 (0.31) ^b^	<0.0001
BMI-based obesity ^†^ (yes)	213 (30.4)	0 (0) ^a^	29 (29) ^a,b^	10 (7.25) ^a,c^	152 (51.18) ^a,d^	<0.0001
Waist Circumference (cm)	98.56 (0.55)	84.09 (0.94) ^a^	96.54 (1.18) ^b^	94.28 (0.95) ^b^	105.95 (0.65) ^c^	<0.0001
Abnormal Waist ^$^ (yes)	560 (79.55)	47 (33.33) ^a^	88 (88.00) ^b^	105 (76.09) ^c^	293 (98.65) ^d^	<0.0001
Blood Pressure						
Systolic (mmHg)	120.79 (0.62)	109.48 (1.23) ^a^	111.14 (1.47) ^a^	123.97 (1.25) ^b^	127.19 (0.85) ^b^	<0.0001
Diastolic (mmHg)	72.94 (0.39)	67.69 (0.82) ^a^	68.94 (0.97) ^a^	74.70 (0.83) ^b^	75.40 (0.57) ^b^	<0.0001
Glucose Homeostasis						
Glucose (mg/dL) (670)	92.02 (0.85)	82.58 (1.79) ^a^	83.43 (2.18) ^a^	93.24 (1.83) ^b^	97.78 (1.24) ^b^	<0.0001
Insulin (mg/dL) (688)	10.25 (0.29)	5.53 (0.53) ^a^	8.35 (0.62) ^b^	7.90 (0.54) ^b^	13.34 (0.36) ^c^	<0.0001
HOMA-IR (662)	2.48 (0.10)	1.12 (0.18) ^a^	1.73 (0.22) ^a,b^	1.83 (0.19) ^b^	3.42 (0.13) ^c^	<0.0001
Lipids						
Total Chol. (mg/dL) (693)	188.49 (1.62)	182.35 (3.56) ^a^	198.42 (4.23) ^b,c^	186.06 (3.67) ^a,c^	190.86 (2.48) ^a,c^	0.0229
HDL-C (mg/dL) (693)	55.88 (0.59)	61.23 (1.24) ^a^	64.73 (1.47) ^a^	52.48 (1.28) ^b^	52.61 (0.86) ^b^	<0.0001
LDL-C (mg/dL) (693)	110.28 (1.33)	103.18 (2.94) ^a^	115.49 (3.50) ^b^	109.04 (3.03) ^a,b^	113.49 (2.05) ^b^	0.0150
TG (mg/dL) (693)	123.44 (2.78)	78.74 (5.61) ^a^	84.93 (6.67) ^a^	141.93 (5.77) ^b^	148.45 (3.90) ^b^	<0.0001
Lifestyle						
Phys. activity score (702)	2.23 (0.02)	2.45 (0.05) ^a^	2.21 (0.06) ^b^	2.47 (0.05) ^a^	2.02 (0.04) ^b^	<0.0001
Current smoker (yes)	104 (14.77)	28 (19.86) ^a^	7 (7.00) ^b^	21 (15.22) ^a,b^	43 (14.48) ^a,b^	0.0619
# Drinks per day (700)	0.49 (0.03)	0.47 (0.06) ^a^	0.41 (0.08) ^a^	0.67 (0.07) ^a,b^	0.44 (0.04) ^a,c^	0.0181
	**Full Sample** **(N = 704)**	**Secondary Dietary Analyses; (N = 613)**				**Overall *p*-Value**
		**MHNW** **(n = 129)**	**MHO** **(n = 92)**	**MUNW** **(n = 193)**	**MUO** **(n = 298)**	
Total calories	1975.28 (32.35)	2080.87 (79.15) ^a^	1883.17 (78.41) ^a^	2040.72 (61.31) ^a^	1920.99 (51.24) ^a^	0.1578
Protein	86.27 (1.46)	90.96 (3.40) ^a^	84.72 (3.76) ^a^	85.11 (2.75) ^a^	85.63 (2.33) ^a^	0.5404
Animal fat	34.24 (0.72)	35.13 (1.84) ^a^	32.08 (1.73) ^a^	34.03 (1.42) ^a^	34.70 (1.10) ^a^	0.6463
Vegetable fat	40.04 (0.97)	42.18 (2.71) ^a^	40.73 (2.36) ^a^	41.01 (1.84) ^a^	38.37 (1.45) ^a^	0.4886
Total fat	74.29 (1.41)	77.31 (3.67) ^a^	72.81 (3.36) ^a^	75.04 (2.66) ^a^	73.08 (2.20) ^a^	0.7245
Carbohydrates	234.77 (4.21)	251.04 (10.24) ^a^	219.84 (10.47) ^a^	244.10 (7.96) ^a^	227.11 (6.64) ^a^	0.0732
Saturated fat	24.41 (0.49)	25.63 (1.26) ^a^	24.07 (1.25) ^a^	24.32 (0.89) ^a^	24.09 (0.76) ^a^	0.7242
Mono-unsaturated fat	28.14 (0.60)	29.22 (1.39) ^a^	27.01 (1.37) ^a^	28.58 (1.18) ^a^	27.78 (0.97) ^a^	0.7256
Poly-unsaturated fat	14.75 (0.32)	15.33 (0.97) ^a^	14.74 (0.82) ^a^	14.89 (0.59) ^a^	14.42 (0.44) ^a^	0.7823

Data are presented as mean (standard error of the mean, SEM) or number of participants (%). Same letter superscript indicates no statistical significance between the groups; different letter superscripts indicate statistically significant difference between the groups. (N = sample size) is specified for variables with missing data (<704), n is used for phenotypic group sample size. Abbreviations: BMI, body mass index; MHNW, metabolically healthy normal weight; MHO, metabolically healthy obesity; MUNW, metabolically unhealthy normal weight; MUO, metabolically unhealthy obesity; HOMA-IR, homeostatic model assessment-insulin resistance; HDL-C, high-density lipoprotein cholesterol; LDL-C, low-density lipoprotein cholesterol; TG, triglycerides. ^†^ BMI-based obesity (BMI ≥ 30 km/m^2^). ^$^ Abnormal waist circumference (>102 cm in men, >88 cm in women).

**Table 2:** Regression coefficients for log-transformed liver fat content and probability of steatosis on metabolic health and obesity phenotypes defined by DXA and having ≥1 metabolic dysfunction(s), adjusted for mixed effects.

	Log-Transformed Liver Fat			Steatosis (Yes)		
	β	SE	*p*-Value	β	SE	*p*-Value
MHNW	Reference			Reference		
MHO	0.291	0.083	0.0005	0.984	0.292	0.0003
MUNW	0.247	0.077	0.001	0.958	0.266	<0.0001
MUO	0.756	0.071	<0.0001	1.855	0.254	<0.0001
Age ^†^	−0.0005	0.002	0.792	−0.006	0.005	0.304
Sex (female)	−0.250	0.071	0.0005	−0.453	0.182	0.011
Age × Sex	0.007	0.003	0.008	0.020	0.007	0.005
Age^2^	0.0004	0.00009	<0.0001	−0.001	0.0003	0.0005
Age^2^ × Sex	0.0001	0.0001	0.352	0.00008	0.0004	0.834
Current smoker	0.040	0.070	0.566	0.032	0.181	0.859
Physical activity ^†^	−0.138	0.038	0.0003	−0.187	0.101	0.058
Alcohol ^$^	0.033	0.031	0.289	0.087	0.073	0.236

Abbreviations: MHNW, metabolically healthy normal weight; MHO, metabolically healthy obesity; MUNW, metabolically unhealthy normal weight; MUO, metabolically unhealthy obesity. ^†^ Mean-centered. ^$^ Alcohol consumption (number of drinks per day).

**Table 3:** Regression coefficients for log-transformed liver fat content and probability of steatosis on metabolic health and obesity phenotypes defined by DXA and having ≥2 metabolic dysfunctions, adjusted for mixed effects.

	Log-Transformed Liver Fat			Steatosis (Yes)		
	β	SE	*p*-Value	β	SE	*p*-Value
MHNW	Reference			Reference		
MHO	0.350	0.060	<0.0001	0.967	0.183	<0.0001
MUNW	0.427	0.097	<0.0001	1.066	0.239	<0.0001
MUO	0.941	0.068	<0.0001	1.825	0.198	<0.0001
Age ^†^	−0.0009	0.002	0.650	−0.004	0.005	0.455
Sex (female)	−0.230	0.068	0.0008	−0.432	0.181	0.015
Age × Sex	0.007	0.002	0.004	0.018	0.007	0.013
Age^2^	−0.0004	0.0001	<0.0001	−0.001	0.0003	0.0002
Age^2^ × Sex	1.599	0.0001	0.216	0.00001	0.0004	0.970
Current smoker	0.072	0.067	0.282	0.114	0.177	0.524
Physical activity ^†^	−0.115	0.036	0.002	−0.124	0.104	0.217
Alcohol ^$^	0.043	0.030	0.157	0.120	0.076	0.105

Abbreviations: MHNW, metabolically healthy normal weight; MHO, metabolically healthy obesity; MUNW, metabolically unhealthy normal weight; MUO, metabolically unhealthy obesity. ^†^ Mean-centered. ^$^ Alcohol consumption (number of drinks per day).

## Data Availability

The data presented in this study are available on request from the corresponding author.

## Supplementary Materials

The following supporting information can be downloaded at: https://www.mdpi.com/article/10.3390/metabo16050299/s1, Supplementary Table S1: Associations of dietary variables with log-transformed liver fat in SOLAR models; Supplementary Table S2: Regression coefficients for log-transformed liver fat content and probability of steatosis on metabolic health and obesity phenotypes defined by BMI and having ≥1 metabolic dysfunction(s), adjusted for mixed effects; Supplementary Table S3: Regression coefficients for log-transformed liver fat content and probability of steatosis on metabolic health and obesity phenotypes defined by BMI and having ≥2 metabolic dysfunctions, adjusted for mixed effects.

## Abbreviations

ALT	Alanine Aminotransferase
AUC	Area Under the Curve
BMI	Body Mass Index
CVD	Cardiovascular Disease
DBP	Diastolic Blood Pressure
DXA	Dual-Energy X-ray Absorptiometry
FFQ	Food Frequency Questionnaire
FG	Fasting Glucose
FLS	Fels Longitudinal Study
HDL-C	High-Density Lipoprotein Cholesterol
HOMA-IR	Homeostatic Model Assessment of Insulin Resistance
HTGC	Hepatic Triglyceride Content
HTN	Hypertension
LDL-C	Low-Density Lipoprotein Cholesterol
MASLD	Metabolic Dysfunction-Associated Steatotic Liver Disease
MHNW	Metabolically Healthy Normal Weight
MHO	Metabolically Healthy Obesity
MRI	Magnetic Resonance Imaging
MUO	Metabolically Unhealthy Obesity
MUNW	Metabolically Unhealthy Normal Weight
NAFLD	Non-Alcoholic Fatty Liver Disease
NFS	NAFLD Fibrosis Score
PCA	Principal Component Analysis
PDF	Proton Density Fat Fraction
SBP	Systolic Blood Pressure
SEM	Standard Error of the Mean
T2D	Type 2 Diabetes
TG	Triglycerides
