# Supplementary material for: Cross-Sectional Associations of Metabolically Healthy Obesity, Lifestyle Factors, and Steatotic Liver Disease in Adults from the Fels Longitudinal Study
Source: Metabolites. 2026 Apr 28;16(5):299. doi: 10.3390/metabo16050299 (PMC13208267; doi:10.3390/metabo16050299)
Supplement: Supplementary file 1 [file metabolites-16-00299-s001.zip › metabolites-4273588-supplementary.pdf]

SUPPLEMENTARY TABLES

Supplementary table 1. Associations of dietary variables with log-transformed liver fat in SOLAR models.

	Log-transformed liver fat		
	β	***SE***	***P-value***
Individual Dietary Exposures			
Total fat	3.990×10^{-4}	9.647×10^{-4}	0.679
Total calories	2.516×10^{-5}	4.303×10^{-5}	0.559
Carbohydrates	9.569×10^{-5}	3.322×10^{-4}	0.773
Saturated fat	3.244×10^{-3}	2.806×10^{-3}	0.247
Animal fat	3.280×10^{-3}	1.915×10^{-3}	0.087
Diet scores			
Liver fat diet score (LFDS)	0.027	0.014	0.064
LFDS x MHO	-0.053	0.049	0.288
LFDS x MUNW	3.191×10^{-4}	0.041	0.993
LFDS x MUO	0.024	0.034	0.539
Dietary PCA patterns			
PC1 (Overall intake)	6.708×10^{-3}	0.012	0.597
PC2 (fat quality)	0.064	0.035	0.069
PC3 (carb-heavy)	-0.030	0.046	0.514
PC x MHO interactions	-	-	All not sig.
PC x MUNW interactions	-	-	All not sig.
PC x MUP interactions	-	-	All not sig.

Abbreviations: MHNW, metabolically healthy normal weight; MHO, metabolically healthy obesity; MUNW, metabolically unhealthy normal weight; MUO, metabolically unhealthy obesity; PCA, principal components analysis; PC1, 2, 3, principal components 1, 2 and 3. All models adjusted for age, sex, and their interactions, metabolic phenotype group, smoking, alcohol, physical activity and mixed effects.

Supplementary table 2. Regression coefficients for log-transformed liver fat content and probability of steatosis on metabolic health and obesity phenotypes defined by BMI and having ≥1 metabolic dysfunction(s), adjusted for mixed effects.

	Log-transformed liver fat			***Steatosis (Yes)***		
	β	***SE***	***P-value***	***β***	***SE***	***P-value***
MHNW	***Reference***			***Reference***		
MHO	0.348	0.116	0.003	0.876	0.307	0.004
MUNW	0.305	0.059	<0.0001	0.854	0.179	<0.0001
MUO	0.895	0.067	<0.0001	1.799	0.190	<0.0001
Age†	0.0006	0.002	0.764	-0.002	0.005	0.725
Sex (female)	-0.073	0.065	0.261	-0.069	0.162	0.672
*Age*Sex*	0.007	0.002	0.003	0.018	0.007	0.012
Age^2	-0.0004	0.00009	<0.0001	-0.0009	0.0003	0.0007
Age^2**Sex*	0.0001	0.0001	0.372	-0.00001	0.0004	0.982
Current smoker	0.014	0.067	0.830	-0.012	0.174	0.944
Physical Activity†	-0.153	0.036	<0.0001	-0.199	0.097	0.036
Alcohol$	0.030	0.030	0.316	0.084	0.072	0.246

Abbreviations: MHNW, metabolically healthy normal weight; MHO, metabolically healthy obesity; MUNW, metabolically unhealthy normal weight; MUO, metabolically unhealthy obesity.

† Mean centered

$ Alcohol consumption (number of drinks per day).

Supplementary table 3. Regression coefficients for log-transformed liver fat content and probability of steatosis on metabolic health and obesity phenotypes defined by BMI and having ≥2 metabolic dysfunctions, adjusted for mixed effects.

	Log-transformed liver fat			***Steatosis (Yes)***		
	β	***SE***	***P-Value***	***β***	***SE***	***P-Value***
MHNW	***Reference***			***Reference***		
MHO	0.487	0.070	<0.0001	1.073	0.174	<0.0001
MUNW	0.502	0.068	<0.0001	1.011	0.167	<0.0001
MUO	0.974	0.065	<0.0001	1.695	0.167	<0.0001
Age†	0.0003	0.002	0.873	-0.0003	0.005	0.947
Sex (female)	-0.071	0.063	0.261	-0.080	0.164	0.619
*Age*Sex*	0.007	0.002	0.003	0.016	0.007	0.028
Age^2	-0.0004	0.0001	<0.0001	-0.001	0.0003	0.0001
Age^2**Sex*	0.0001	0.0001	0.231	0.0001	0.0004	0.780
Current smoker	0.045	0.064	0.485	0.082	0.179	0.638
Physical activity†	-0.128	0.035	0.0003	-0.127	0.100	0.190
Alcohol$	0.041	0.029	0.157	0.110	0.073	0.130

Abbreviations: MHNW, metabolically healthy normal weight; MHO, metabolically healthy obesity; MUNW, metabolically unhealthy normal weight; MUO, metabolically unhealthy obesity.

† Mean centered

$ Alcohol consumption (number of drinks per day).
